# 50 Years of the steric-blocking mechanism in vertebrate skeletal muscle: a retrospective

**DOI:** 10.1007/s10974-022-09619-z

**Published:** 2022-07-05

**Authors:** David A. D. Parry

**Affiliations:** School of Natural Sciences, Massey University, Private Bag 11-222, Palmerston North, 4442 New Zealand

**Keywords:** Tropomyosin, Relaxed and contracting muscle, X-ray diffraction, 3-D reconstruction, Troponin, HMM S-1, Actin

## Abstract

Fifty years have now passed since Parry and Squire proposed a detailed structural model that explained how tropomyosin, mediated by troponin, played a steric-blocking role in the regulation of vertebrate skeletal muscle. In this Special Issue dedicated to the memory of John Squire it is an opportune time to look back on this research and to appreciate John’s key contributions. A review is also presented of a selection of the developments and insights into muscle regulation that have occurred in the years since this proposal was formulated.

## Introduction

The steric-blocking mechanism proposed in 1973 by John Squire and the author (Parry and Squire 1973) explained how the regulation of vertebrate skeletal muscle might be facilitated by the combined action of tropomyosin and troponin. Since 50 years have now passed since this research was undertaken (1971/1972) it seems an opportune time to look back on the events of the day that led to those ideas and to the interactions between the authors that contributed to the success of this venture. It is also of interest to consider a selection of the most important developments in this field that have occurred in subsequent years.

In order to put these events in context it may be pertinent to provide here a brief account of the personal history of both authors since this is clearly related to their ability to have collaborated so successfully on the steric-blocking mechanism and, indeed, on many other matters over the years. I first met John Squire in 1963 when he was 18 years old and in his first year of a physics degree at King’s College London (KCL). In turn, I was in my first year of a PhD in biophysics, also at KCL. In order to gain a little income, I undertook some student demonstrating in first year physics laboratory classes and this proved to be the occasion of our first meeting. When my PhD studies were completed under the supervision of Arthur Elliott, it was John Squire who sat down at “my” desk to undertake his own PhD under the same supervisor. This was the second in what proved to be a series of intersections in our respective careers that were destined to play a major part in both of our lives.

During my postdoctoral fellowship with Bruce Fraser and Tom MacRae at the CSIRO Division of Protein Chemistry in Melbourne, Australia, where I worked primarily on keratin structures, I honed my skills in fibre diffraction and model building, both of which were, with hindsight, to play a part in our subsequent research on the steric-blocking mechanism. After Melbourne I spent 2 years (1969–1971) with Carolyn Cohen and Don Caspar at the Childrens Cancer Research Foundation in Boston working on the crystal structures of tropomyosin. Tropomyosin crystals illustrated that dynamic interactions in muscle were probable and that 40 nm long tropomyosin molecules were firmly bonded end-to-end to form an open meshwork of supercoiled filaments (unit cell diagonal 40.2 nm with a variation of only 0.2%). Our work on the molecular rearrangements of tropomyosin in the crystal lattice caused by troponin also illustrated that in vivo some movement of tropomyosin in the thin filaments of muscle was likely (Cohen et al. 1971, 1972).

After John completed his PhD he took up a post-doctoral fellowship in Jack Lowy’s laboratory in Aarhus, Denmark. The main themes of the research undertaken there were X-ray diffraction studies on relaxed and contracting muscle (Lowy 1972; Vibert et al. 1971, 1972) and the structural basis of contraction in muscle (Small and Squire 1972; Squire 1972). Back in the 1960s and early 1970s the use of relatively low intensity X-ray sources, allied to the need to take an X-ray pattern of contracting muscle only for the fraction of a second that the muscle was stimulated to contract, made the total exposure a very prolonged process. Subsequently, however, the use of high intensity sources generated from synchrotron radiation became commonplace and total exposure times were much reduced. A key observation in the early 1970s was that X-ray studies on contracting vertebrate skeletal muscle (Huxley 1970, 1972) and those in rigor (Vibert et al. 1972) showed significant intensity differences on the 2nd and 3rd layer lines of the actin diffraction pattern. These data, allied to the wide knowledge of every aspect of muscle structure and function that John had gained in both the UK and Denmark, were to prove invaluable in our subsequent collaborative venture.

In late 1971 I gained a post-doctoral fellowship to work with Andrew Miller at the Laboratory of Molecular Biophysics in the Department of Zoology. Oxford. At the same time John returned from Denmark and took up a post-doctoral Fellowship with Belinda Bullard, also in the Department of Zoology. Our careers thus intersected for the third time and it soon became apparent that our complementary experiences and expertise might allow us to model the mechanism by which vertebrate skeletal muscle was regulated in vivo. Importantly, and prior to us arriving in Oxford, we had independently come to the conclusion that the movement of tropomyosin in the thin filament was the key factor in regulation.

It is important to acknowledge from the outset that there were many relevant and recently published/in press observations that provided key inputs into our analyses. For example, it had been shown that seven actin monomers were stoichiometrically related to a single tropomyosin molecule (Ebashi and Endo 1968). It had also been shown that the axial period of tropomyosin in muscle measured by X-ray fibre diffraction was 38.5 nm (Huxley and Brown 1967) but that the length of tropomyosin deduced from the crystal studies was about 41.0 nm when supercoiling was removed. Further, O’Brien et al. (1971) had suggested that the X-ray observations on contracting and relaxed muscles might be explicable in terms of changes in the position of tropomyosin in the long-period grooves of the actin helix, though they gave no detailed analyses to support their ideas. However, optical diffraction studies that they undertook on electron micrographs of paracrystals of F-actin and also of thin filaments containing tropomyosin (and sometimes troponin) indicated an enhancement of the 2nd layer line diffraction when tropomyosin was present. They did not, however, indicate what structural changes might occur in the thin filaments during muscle contraction when tropomyosin was always present. Another key observation was that derived from the 3-D reconstructions of Moore et al. (1970) on actin filaments. These showed the sites where the heads of myosin, in the absence of ATP, bound to the actin filaments. These were all to prove crucial pieces of evidence in our model-building process as were the X-ray data on relaxed and contracting muscle pertaining to the 2nd and 3rd layers lines of the actin diffraction pattern previously noted.

Thus, although the general form of thin filament structure was well defined in the early 1970s no detailed analyses of the structural changes that might occur in the thin filaments during muscle regulation had been undertaken. This was the point from which our own research efforts were to commence and which were to climax, in a remarkably short time thereafter, in a model that has very largely withstood the passage of time.

### Structural analyses

Significantly, in the steric-blocking mechanism paper (Parry and Squire 1973) the first topic that we discussed related to the Mg tactoids of tropomyosin (Caspar et al. 1969: axial period 39.5 nm): it was noted that these displayed 14 more-or-less equally-separated axial bands. This suggested that the tropomyosin sequence might contain a quasi-repeat of approximately this magnitude, i.e. 39.5/14 or 2.82 nm, a value equivalent to 2.82/0.1485 or 19 residues in a coiled-coil conformation of the type adopted by tropomyosin. This was virtually identical to half the separation of consecutive actin molecules in the thin filament and corresponded directly to the 2.8 nm meridional reflection seen earlier by X-ray diffraction (Caspar et al. 1969). Although the complete sequence of tropomyosin was unknown in 1971 the idea that there might be a close correspondence between the period in tropomyosin and that in the actin helix was very suggestive to us that periodic interactions between tropomyosin and actin would occur, a concept consistent with tropomyosin lying in any of a number of positions in the grooves of the actin-containing thin filaments.

In addition, we showed that if the molecules of tropomyosin (length 41.0 nm) were supercoiled with a radius of about 3.0 nm, as would be likely in the grooves of the actin helix, the axial period would be 39.5 nm, a value akin to the observed period of 38.5 nm. Any additional supercoiling of tropomyosin would necessarily reduce the 39.5 nm value to one closer to 38.5 nm and, indeed, it was shown that a supercoil of pitch 5.5 nm and radius 0.25 nm would be sufficient to take up the entire length of the molecule.

As noted earlier X-ray diffraction data (Huxley 1970, 1972; Vibert et al. 1972) had shown that in relaxed vertebrate skeletal muscle the intensity on the 2nd layer line of the actin pattern (I_2_) was less than that on the 3rd layer line (I_3_, where I_2_ < I_3_). In contrast, in contracting muscle the intensity on the 2nd layer line was considerably greater than that in relaxed muscle (i.e. > I_2_) and that on the 3rd layer line was smaller (i.e. < I_3_). As a result the 2nd layer line intensity was greater than that on the 3rd layer line. This was a key observation and one that strongly informed our model-building studies. Indeed, very early model calculations showed that the second layer line of the actin pattern was particularly sensitive to the position of tropomyosin. We now found ourselves to be in a position to undertake detailed model-building and to calculate the diffraction pattern of models with tropomyosin lying in different positions in the long-period grooves of the thin filament. We hoped that we might be able to match the qualitative experimental observations noted above.

Computers in the early 1970s were rather primitive by present-day standards. This necessitated a suitably simple model amenable to ready calculation. Consequently, the symmetry of the thin filament was modelled as that of a 13/6 helix with an axial repeat of 35.5 nm. Each actin molecule was represented by a sphere of radius 2.4 nm and the length of tropomyosin associated with each actin (5.5 nm) was modelled as five overlapping spherical scattering units, each with a radius of 0.83 nm. Each of these was separated from its immediate neighbour by an axial distance of 1.1 nm. The radius of 0.83 nm arose from the need to ensure that the ratio of the combined volumes of the five scattering units representing tropomyosin to that of the volume of the actin sphere matched the ratio of one-seventh of the molecular weight of tropomyosin to the molecular weight of actin. This, in turn, was dictated by the fact that the electron densities of tropomyosin and actin were virtually identical (430–440 el nm^−3^).

**Fig. 1:**
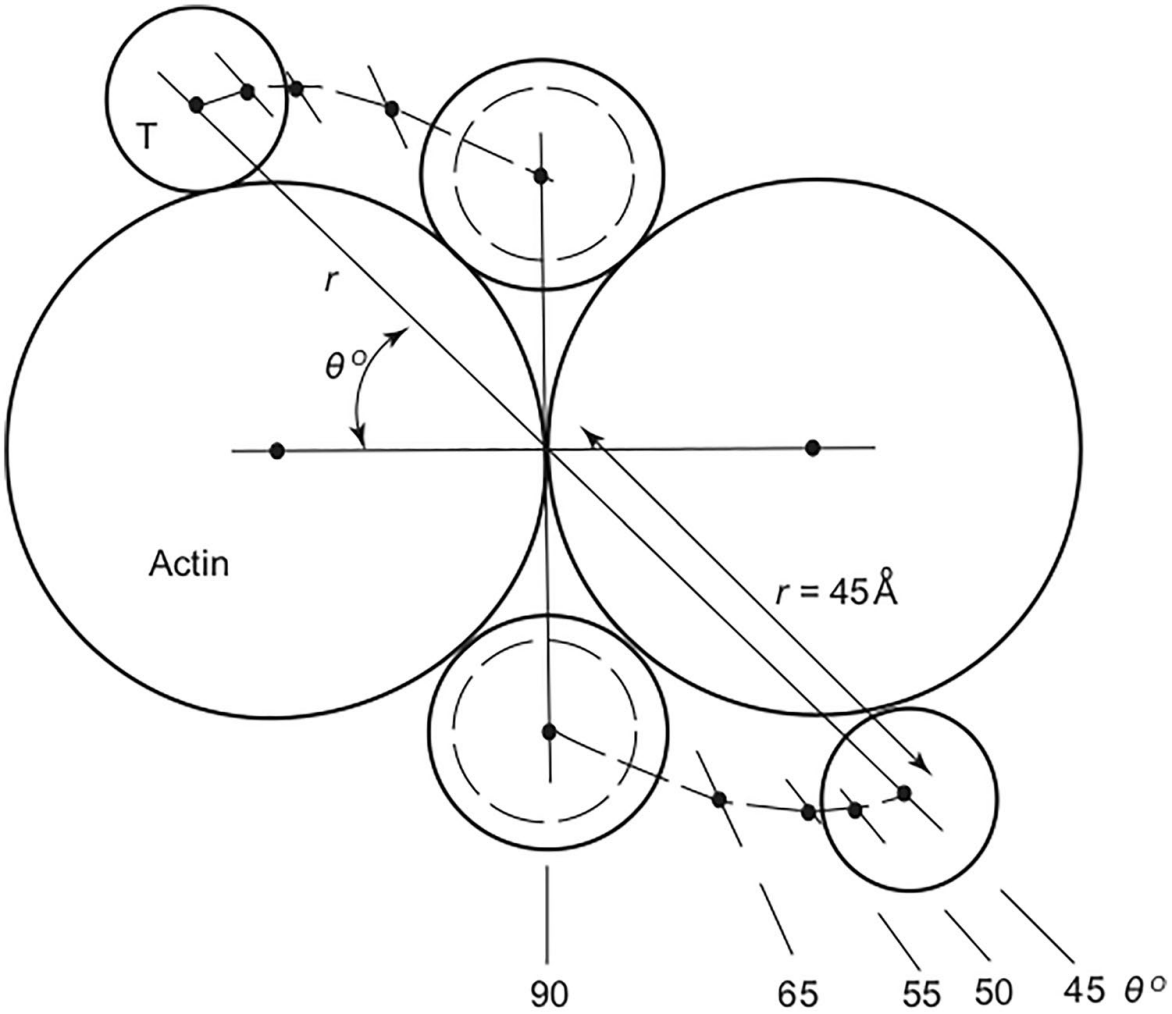
Thin filament in cross-section with the two long-period actin strands considered as continuous threads. The position of tropomyosin (T), assuming that it lies on the surface of actin, is defined by radial coordinates (*r*, *θ*), where *θ* is allowed to vary between 45° and 90°. For larger values of *θ* it is believed that the tropomyosin molecules will display a degree of supercoiling. Redrawn from Parry and Squire (1973)

**Fig. 2:**
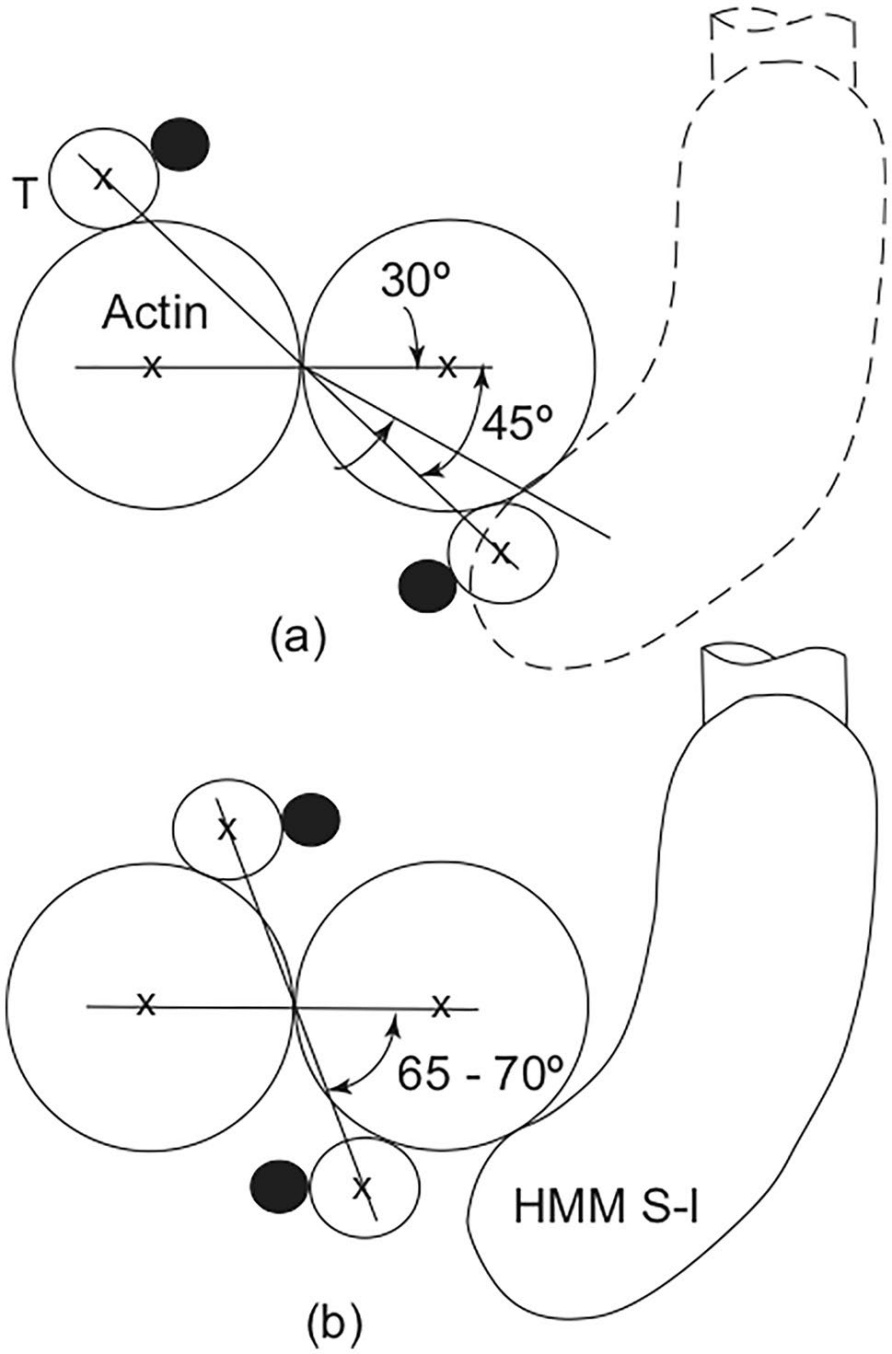
Axial projection of the thin filament showing tropomyosin (small circles), troponin (filled circles), actin (large circles) and HMM S-1. **a** corresponds to relaxed vertebrate skeletal muscle with the position of tropomyosin sterically-blocking the attachment of HMM S-1 to actin and **b** contracting vertebrate skeletal muscle with tropomyosin in a position closer to the centre of the central groove thereby permitting HMM S-1 to bind to actin. Redrawn from Parry and Squire (1973)

The Fourier transforms of ten models of the thin filament were thus calculated. These differed only in the azimuthal position of tropomyosin (Fig. 1), which was systematically rotated in 5° intervals about the long axis of the thin filament from a point where it lay central in the groove formed by the two long-period strands of actin monomers (azimuth 90°) to one where it was well displaced from it (azimuth 45°). The calculations were striking and immediately informative: the intensity on the second layer clearly increased with increasing azimuth whereas the intensity on the third layer line decreased with increasing azimuth. This strongly indicated that if tropomyosin was to move from a position with azimuth 45–50° (radius about 4.2–4.5 nm) in relaxed vertebrate skeletal muscle to a position with azimuth 65–70° (radius 3.3–3.5 nm) in contracting muscle the observed intensity changes on the 2nd and 3rd layers of the actin pattern would be very satisfactorily explained. In addition, in relaxed muscle the tropomyosin filaments would lie in very close proximity to the known binding site of the HMM S-1 (head) fragment of myosin on actin and could thereby sterically-block myosin from interacting with actin. In contrast, in contracting muscle the position of the tropomyosin filaments would be displaced from the HMM S-1 binding site and would not hinder actomyosin interaction. If nothing else the steric-blocking concept was striking in its simplicity and was, we felt, particularly attractive because of it (Fig. 2).

**Fig. 3:**
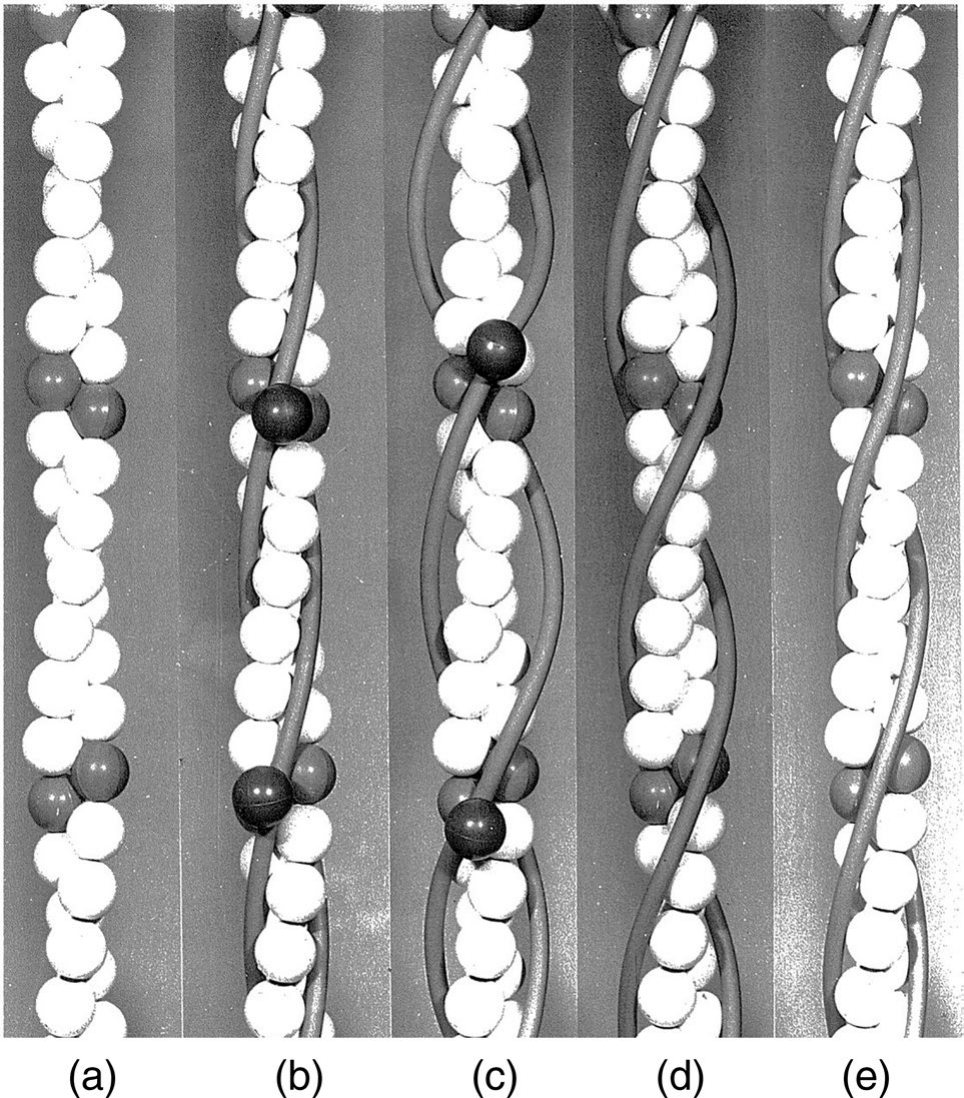
**a** Model of F-actin with 13/6 symmetry, approximate radius 2.4 nm and repeat length 35.5 nm. The actin molecules are represented by white balls although every seventh one along a single long-period strand is represented by a red ball (it appears light grey) to illustrate the functional unit, which comprises one tropomyosin molecule, seven actin molecules and one troponin molecule. The latter, placed in an arbitrary position, is represented by a dark green ball (it appears dark grey); **b** Tropomyosin is shown tightly bound in the centre of the long-period grooves; **c** tropomyosin is shown attached at axial intervals of 38.5 nm. This illustrates that the binding between actin and tropomyosin would be very loose unless tropomyosin was supercoiled; **d** tropomyosin is shown bound to actin with an azimuth of 45° (see text). Tropomyosin would sterically-block the attachment of the myosin head to actin and this would correspond to muscle in a relaxed state; **e** tropomyosin is bound to actin with an azimuth of 65°. Tropomyosin is now in a position that would permit binding of the myosin head to actin and would correspond to muscle in a contracting state. Reproduced from Parry and Squire (1973) with permission from Elsevier

A physical model was constructed by John to illustrate the position of tropomyosin filaments in the “on” and “off” states in the actin thin filaments. About 40 rubber balls were purchased from Woolworths in the High Street in Oxford—white, red and dark green. John felt slightly guilty about cleaning out the stock of rubber balls from the store and thereby depriving the local children of their entertainment but the needs of science prevailed. Each actin was represented by a white ball but every seventh one was replaced by a red one, thereby indicating the extent of the “structural unit”. Troponin was represented by a dark green ball. A hole was drilled through the centre of the “actin” balls and then each was threaded on to a thin wire, thereby representing one of the two long-period strands comprising the actin filaments. The tropomyosin filaments were represented by lengths of rubber tubing “borrowed” from the chemistry laboratories. The resulting figure in our paper showed various positions of tropomyosin in the long period grooves of the thin filament, and was successful in illustrating to readers exactly what the steric-blocking mechanism implied (Fig. 3). Nowadays, the model would be computer-generated and would be much more attractive visually. It nonetheless served its purpose well at the time.

Progress on the steric-blocking project was very rapid indeed from its inception in early November 1971 to the time when I gave a seminar on the completed model to the Department of Zoology, Oxford on 21 January 1972. A few months of tidying up the manuscript followed before it was submitted on 9 May 1972 and published in 1973. In a note added in proof we commented that after our paper had been submitted we learnt that Hugh Huxley and John Haselgrove were carrying out similar calculations. They subsequently published these in the Proceedings of the 1972 Cold Spring Harbor Symposium on Quantitative Biology (Huxley 1972; Haselgrove 1972) and, indeed, their conclusions closely matched our own.

John and I agreed that we had contributed equally to the 1973 paper. However, unlike today where an asterisk would be placed by both author’s names indicating equal contributions, we decided to toss a coin to decide whether it should be Parry and Squire (“heads”) or Squire and Parry (“tails”). John tossed the coin and thus the order was decided. It made no real difference to either of us, of course, and we always received equal recognition for our efforts. This represented the only time in my scientific career when the order of authors on a paper I contributed to was decided by the toss of a coin.

It is worth noting that the results presented were not confined to the regulation of vertebrate skeletal muscle but included those for both molluscan muscle and vertebrate smooth muscle. Furthermore, it was shown that the approximately 20% increase in intensity observed on both the 5.1 and 5.9 nm layer lines of the actin pattern as muscles go from a relaxed to a contracting state (Haselgrove 1970; Lowy 1972; Vibert et al. 1972) was not a consequence of changes in either the structure of actin or in the organisation of the tropomyosin filaments but arose from cross-bridge attachment. Nowadays there is a wealth of information supporting that conclusion, of course, but in the early 1970s this was not the situation and the idea was considered likely but not unequivocal. Looking back at the 1973 paper it was interesting to see the lengths we went to in order to convince ourselves (and others) that the only realistic solution to the intensity changes observed on the 2nd and 3rd layer lines between the relaxed and contracting states was that involving the movement of tropomyosin.

With hindsight everything came together far more successfully than we could have ever imagined. The data on which we based our ideas were very limited: perhaps we were fortunate in getting things right but maybe we just spotted the obvious interpretation. Either way this paper was to give both of us one of the greatest thrills of our scientific careers and was one that we looked back on in subsequent years with a great deal of affection, not least because this represented the first opportunity we had been afforded to work closely together.

### Later research

Any scientifically-significant paper, as we hoped our 1973 paper might prove to be, should not only advance the field but should also provide the framework for future development. Our desire, therefore, was that our contribution would represent a new beginning in the regulation story and one that would facilitate the gaining of new insights into the mechanism.

As is often the case with a new concept the regulatory model, as originally presented, was not universally accepted and, indeed, it often proved to be the source of considerable debate and the expression of fervently-held views. This, of course, was entirely appropriate and represented the way that any scientific enterprise worthy of the name should be addressed. While details of the controversies are not described here (see, however, Squire and Morris (1998) for a discussion of many of the relevant issues) this does not lessen the importance of the role that they played in the future development of the regulatory mechanism.

Subsequent progress by ourselves (primarily John Squire, Ed Morris, Danielle Paul and their colleagues) but also by others (including Peter Vibert, Bill Lehman, Roger Craig, Carolyn Cohen, Keiichi Namba and Takashi Fujii and their colleagues) has very much confirmed those hopes. It is not the purpose of this paper to review all of the progress on the regulation of vertebrate skeletal muscle that has been made since 1971/1972 when this research was undertaken—now 50 years ago. Rather, a small number of areas of particular interest to the author, primarily relating to structural/functional aspects, have been selected and these are discussed below. These have either confirmed the essence of the steric-blocking mechanism or have given rise to exciting new insights. Much more detailed accounts of these and other significant developments are provided in the reviews by Squire et al. (2017) and Hitchcock-DeGregori and Barua (2017).

As noted earlier the Mg tactoids of tropomyosin (axial period 39.5 nm) displayed 14 more-or-less equally-separated bands. This suggested that the sequence might contain a quasi-repeat of about 2.82 nm. Subsequent sequence analyses (Parry 1974, 1975; McLachlan and Stewart 1976) did indeed show that the sequence of rabbit alpha-tropomyosin (284 residues) had a repeat of about 39.2 residues (5.8 nm) that was strongly halved (19.6 residues or 2.9 nm) in the axial distributions of both the acidic residues and the apolar residues, and that approximately eight residues were involved in a head-to-tail overlap of similarly-directed tropomyosin molecules in the filaments. These periods were easily and directly related to the separation of actin monomers along one strand of the thin filaments. It followed naturally that although the seven (14) repeats were not identical to one another it was indeed possible for each actin to be regulated by tropomyosin in a quasi-equivalent manner closely akin to that embodied in our proposals.

Using 3-D reconstruction methodology on electron micrographs of thin filaments Spudich et al. (1972) showed that tropomyosin filaments sometimes lay to one side of the centre of the actin grooves in a position close to one of those that we had identified. Interestingly, however, helical reconstructions of various muscles in different states (Lehman et al. 1994, 1995; Vibert et al. 1997; reviewed by Squire et al. 2017) revealed that tropomyosin filaments were not found in just two positions (on and off) on the actin filament, as we had suggested, but in three positions. The first, when the calcium levels were low, was termed the “off” position (the blocked or B-state), and corresponded to the situation where the attachment of the myosin heads was almost completely blocked. The second corresponded to the so-called intermediate state (the closed or C-state), which resulted from calcium activation of thin filaments, where the tropomyosin filaments were rotated a further 20° relative to that in the off state. The third state (the myosin or M-state) occurred when the myosin heads were bound strongly. In this case the tropomyosin filaments were rotated a further 10° relative to that in the C-state. Over the years a considerable body of evidence has been accumulated that supports these conclusions (see, for example, Phillips et al. 1986; McKillop and Geeves 1993; AL-Khayat et al. 1995; Brown and Cohen 2005; Poole et al. 2006) and, together, they represent a significant development in our understanding of regulation.

Dividing electron microscope images of filaments into short segments has allowed three-dimensional reconstructions to be performed at much higher resolutions than were previously thought possible. Notable amongst the successes using this technique are those for actin filaments (resolution 0.66 nm, Fujii et al. 2010), actin-tropomyosin (resolutions 0.37 nm for F-actin and 0.65 nm for tropomyosin, van der Ecken et al. 2015) and actin-tropomyosin-myosin in the rigor state (resolution 0.8 nm, Behrmann et al. 2012). Between them these structures (and refined versions of some of them) have provided a number of fascinating insights. For example, it has become clear that interactions between actins along an individual long-pitched strand are strong whereas those interactions between the strands are relatively loose. Further, in the Behrmann et al. (2012) structure the interactions between a single tropomyosin sub-repeat, two neighbouring actins along a long-period strand and a myosin head (in the rigor state) revealed, as predicted, that the tropomyosin lay very close to the M-state and, furthermore, that the tropomyosin strands were interacting tightly with the myosin heads. Many important details of the steric-blocking mechanism have been demonstrated by studies of this type and these have proved invaluable in the on-going increase in our understanding of the steric-blocking mechanism.

It is always exciting when something unexpected turns up. In single particle image processing of negatively-stained thin filaments in the absence of calcium (Paul et al. 2017) tropomyosin was shown to lie in essentially equivalent positions on each actin in the thin filament. This, in itself, was no surprise, of course, as it had previously been thought likely because of the quasi-equivalence of the seven sets of sequence repeats in tropomyosin, the earlier helical reconstructions from electron micrographs and also the X-ray diffraction data, all of which tend to “see” average structures rather than ones displaying local variation. In the presence of calcium, however, the situation was rather different. For ease of explanation of the results, the seven quasi-repeats in the sequence of tropomyosin were labelled as *a*, *b*, *c*, *d*, *e*, *f* and *g*, where *a*, *b* and *c* constitute what has been termed as set 2 and where d, *e*, *f* and *g* constitute what has been termed as set 1 (Squire et al. 2017). The validity of sub-dividing the tropomyosin repeats in this manner relies on the evidence presented by Paul et al. (2017) that the negative staining of their specimens, allied to the resolution limits of their data, does indeed allow a clear differentiation to be made between troponin subunits and tropomyosin along the length of the thin filaments. On this basis Paul et al. (2017) have suggested that the tropomyosin repeats comprising set 1, which are close to troponin on the pointed/M-line end of the thin filament, shift across the filament by about 18° but the tropomyosin repeats that comprise set 2, and which lie on the other side of troponin at the barbed/Z-line end of the thin filament, move by an average of about 28°. Thus, in the presence of calcium the set 1 repeats of tropomyosin lie in the closed C-state whereas the set 2 tropomyosin repeats, even in the absence of myosin, lie in a position that is close to the M-state. It follows that tropomyosin does not move as a rigid body and that the lateral shift of the tropomyosin repeats can show variation from actin-to-actin such that some myosin sites on actin may be completely open and some may be partially closed (Paul et al. 2017).

Using cryo-electron microscopy, which preserves proteins in a near-native frozen hydrated state, single particle image analysis has recently broken the sub-nanometer barrier, with detailed structures of the thin filaments in cardiac muscle, both in the presence and absence of Ca^2+^ (Yamada et al. 2020). In conjunction with known crystal structures this has revealed that the head-to-tail overlap of tropomyosin molecules lies in a complex with an N-terminal region of troponin T and a C-terminal region of troponin I. Further, these studies have shown the core of troponin lying on the actin filament. The regulatory mechanism has thus been explained as follows: in the absence of Ca^2+^ the C-terminal part of troponin I binds to both actin and that part of tropomyosin that lies above the troponin core. Consequently, the HMM S-1 binding sites on actin are blocked. However, when Ca^2+^ is bound by a region in the N-terminal part of troponin C the complete C-terminal fragment of troponin I dissociates from the complex by the binding of a short N-terminal sequence in the C-terminal part of troponin I to a region in the N-terminal fragment of troponin C. This allows tropomyosin and the N-terminal portion of troponin T that lies near the head-to-tail junction of the tropomyosin molecules to move across the surface of actin, thereby revealing some of the HMM S-1 binding sites on actin (Yamada et al. 2020). This research has enabled us to gain a much more detailed understanding of the regulatory mechanism at the molecular level than was previously possible.

More progress on the troponin structure was reported for murine cardiac thin filaments using the technique of cryo-electron microscopy (Oda et al. 2020). By incorporating a Volta phase plate the contrast in the micrographs was enhanced considerably and Oda et al. (2020) were also able to visualise a complete repeat unit in the thin filament. Also, in 2021 Risi et al. published a series of cryo-EM maps of the cardiac thin filament at physiological Ca^2+^ levels, where the two strands consist of a mixture of regulatory units, composed of Ca^2+^-free, Ca^2+^-bound or, as observed for the first time, “mixed” with Ca^2+^-bound on one side and Ca^2+^-free on the other.

In yet another recent development Wang et al. (2021) used electron cryo-tomography to investigate structural details of the various regions comprising the mouse sarcomere in the rigor state. Amongst the results reported were the I-band structures of the actin-tropomyosin-troponin complex (resolution 1.98 nm) and the actin-tropomyosin complex (resolution 1.06 nm). After a variety of image processing/refinement steps these authors were able to demonstrate that tropomyosin in the thin filaments in the Ca^2+^ state did indeed lie in the C-state. However, in the A-band the data clearly indicated that tropomyosin lay in the M-state. It confirmed earlier work that the position of tropomyosin on actin can differ locally within the same filament and also within the same sarcomere (Paul et al. 2017). Wang et al. (2021) also showed that there were considerable similarities between skeletal and cardiac troponin when bound to actin filaments.

**Fig. 4:**
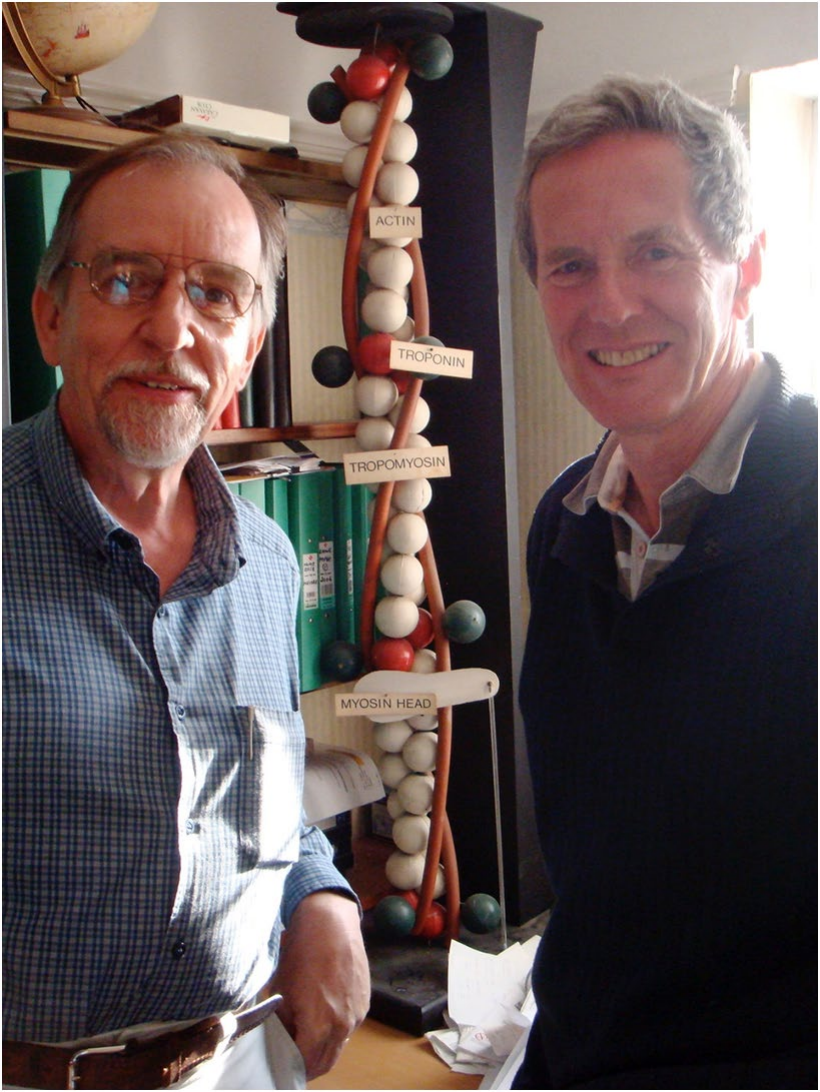
On a visit by the author to the UK John Squire (left) and the author (right) are pictured in the former’s study in Salisbury, England with the steric-blocking model in the background (see Fig. 3)

All of these results, and there are other important ones not described here, have confirmed the essence of the steric-blocking mechanism proposed in 1973. Of course, refinements and new data have altered some aspects and thereby revealed many of the intricacies and details of the regulatory mechanism in vertebrate skeletal muscle that could not have been imagined in 1971/1972 with the evidence then available. When John and I last met at the end of 2019 we still looked back on this project with some pleasure and rejoiced that it had not only survived but had evolved and thrived. The rubber ball model constructed 50 years ago remained with John, at work or in his home study, over this entire period and remained a permanent memento of an exciting scientific time for the pair of us (Fig. 4).

## John Squire’s contributions to the scientific world

John was internationally recognised for his pioneering research in muscle (especially thick filament structure and thin filament regulation), but also in other areas too, such as the glycocalyx, and he published in excess of 100 peer-reviewed papers in the international literature as well as 36 reviews, some in books and some in Journals. Although John and I published only six papers together over a span of 44 years we remained in close contact (person-to-person, mail, fax, phone or email depending on the era) from 1963 until his untimely and tragic passing in January 2021. My wife Jenny and I visited John and Melanie in Salisbury late in 2019 just before “covid” became a word that the world would rather forget. We look back at our last visit with much pleasure but also, in light of subsequent events, with much sadness.

In addition to his numerous scientific achievements John and I collaborated and organised five four-yearly Workshops at Alpbach in Austria starting in 1993 and finishing in 2009. These were on “Coiled-coils, Collagen and Co-proteins” and were essentially devoted to the structure and function of fibrous proteins. On the completion of the 2009 Workshop we passed the organisation of future meetings on to Andrei Lupas and Dek Woolfson. After each Workshop (except the first) we co-edited Special Issues of the Journal of Structural Biology (we were both on the Editorial Board) that covered the papers presented, thereby providing a permanent record of the advances made. In addition, we co-edited four books, three in the Advances in Protein Chemistry series (“Fibrous Proteins: Coiled-coils, Collagen and Elastomers”, “Fibrous Proteins: Muscle and Molecular Motors” and “Fibrous Proteins: Amyloids, Prions and Beta Proteins”, the latter in conjunction with Andrey Kajava) and, most recently, in 2017 a volume entitled “Fibrous Proteins: Structures and Mechanisms”. John edited two other books as well. He did, of course, also write a highly regarded monograph on muscle in 1981 entitled “The Structural Basis of Muscular Contraction”, and this remains a classic in the field. A second monograph appeared in 1986 entitled “Muscle: Design, Diversity and Disease”. With regard to his service to the scientific community John has no peer. Just as importantly (perhaps even more importantly), John remained a gentleman (an old-fashioned word but very relevant in his case), a true friend to his collaborators, a mentor to his students, and a family man in every respect. Each of us will miss him greatly but his work and contributions to our own experiences will remain with us each and every day. Our lives have been much enriched by his presence.

## Personal footnote

I am reminded of a quote from Dr Seuss that seems particularly pertinent with respect to John’s life and career.

Don’t cry because it's over. Smile because it happened

The sentiments thus expressed would, I suspect, be very much in line with John’s own philosophy. Over a period of some 58 years we enjoyed a close and mutually beneficial relationship at both the personal and scientific level. I consider myself to be very fortunate in both respects, and I deem it a great honour to have been invited to make a contribution to this special issue dedicated to the memory of a great biophysicist, a great family man and, above all, a great friend.
